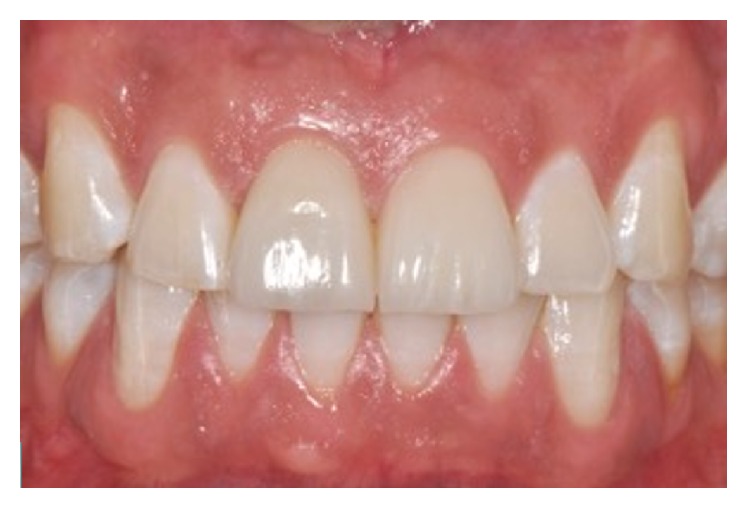# Autogenous Tooth Fragment Adhesive Reattachment for a Complicated Crown Root Fracture: Two Interdisciplinary Case Reports

**DOI:** 10.1155/2016/9352129

**Published:** 2016-11-14

**Authors:** Antonello Francesco Pavone, Marjan Ghassemian, Manuele Mancini, Roberta Condò, Loredana Cerroni, Claudio Arcuri, Guido Pasquantonio

**Affiliations:** ^1^Department of Clinical and Translational Medicine, University of Rome “Tor Vergata”, Rome, Italy; ^2^University of Sydney, Sydney, NSW, Australia; ^3^Catholic University of the Sacred Heart, Rome, Italy; ^4^Unit of Oral Surgery and Implant-Prosthetic Rehabilitation, Rome, Italy

## Abstract

Trauma of anterior teeth is quite a common occurrence in both children and adults. Various degrees of trauma leading to fracture may affect teeth in different ways depending on the age of the patient and extent of fracture and other factors that will be discussed. Guidelines have been given as to how each of these situations should be treated. In the past, often more aggressive restorations were performed to restore fractured teeth. However improved and more efficient adhesion may affect the type of treatment we decide to carry out, leading to more conservative therapies through an increased preservation of tooth structures.

## 1. Introduction

Traumatic injuries to teeth and their supporting tissues usually occur in young people aged 6–13 years, and damage may vary from enamel fracture to avulsion, with or without pulpal involvement or bone fracture [1]. A crown root fracture (CRF) is a type of dental trauma, usually resulting from a horizontal impact, which involves enamel, dentin, and cementum, often occurring below the gingival margin and depending on whether pulp involvement is present or absent, which may be classified as complicated or uncomplicated [2]. Most of these injuries occur in permanent maxillary incisors before complete root formation and cause pulp inflammation or necrosis [1, 2]. Treatment of complicated crown root fractures is often challenging due to difficulty in achieving isolation with a rubber dam for a dry operating field, which might compromise the hermetic seal. Furthermore, dentoalveolar trauma during the maturation of permanent teeth may result in incomplete root formation [3–6]. The nature and depth of the fracture will often dictate the type of treatment that is required. In order to provide predictable esthetics, function, structure, and biologic health, it is imperative that an interdisciplinary treatment approach is followed. This is especially true if the fracture extends into the attachment apparatus or below the osseous crest [4]. The first question the clinician must consider when treatment planning the traumatic fracture is whether the tooth/teeth can be saved. If the fracture extends so far apically that whichever treatment is provided, the resulting crown-to-root ratio is unfavorable, or the amount of coronal tooth structure will not allow restoration, extraction of the tooth and placement of an endosseous implant remains the treatment of choice. The use of natural tooth fragments is an excellent biological approach for restoring fractured anterior teeth [7], when the fragment is available, especially since adhesion technology has improved [8, 9] and further loss of tooth structure can be avoided [10, 11]. Biological restoration using autogenous tooth fragment requires minimal healthy tooth preparation, is esthetic and faster than a complete composite restoration [12], and has a psychological benefit to the patient that his own tooth has been retained [13]. This article addresses the available treatment alternatives when treating complicated fractures. In addition, a step-by-step guide to decision making in order to provide the most predictable results is presented. Two cases will be illustrated using an adhesive approach for the restoration when the tooth is in violation of biologic width to avoid further loss of tooth structure.

## 2. Epidemiology of Fractures

Anterior teeth fractures as a result of traumatic injury are frequently seen in dental practice. A high prevalence is noted in children between 7 and 12 years of age [14, 15]. Often maxillary anterior teeth are affected, and of them 80% are maxillary central incisors, followed by maxillary lateral incisors and mandibular incisors [14, 15]. Average incidence of injuries to anterior teeth reported in literature ranges from 4 to 46%, with 11 to 30% in primary dentition and 6 to 29% in permanent dentition. Epidemiological statistics revealed that crown root fractures represent 5% of dental injuries [14, 15], and the main causes of dental injuries are falls and collisions, sporting activities, violence, and traffic accidents.

## 3. Classification of Fractures

**Figure 1:**
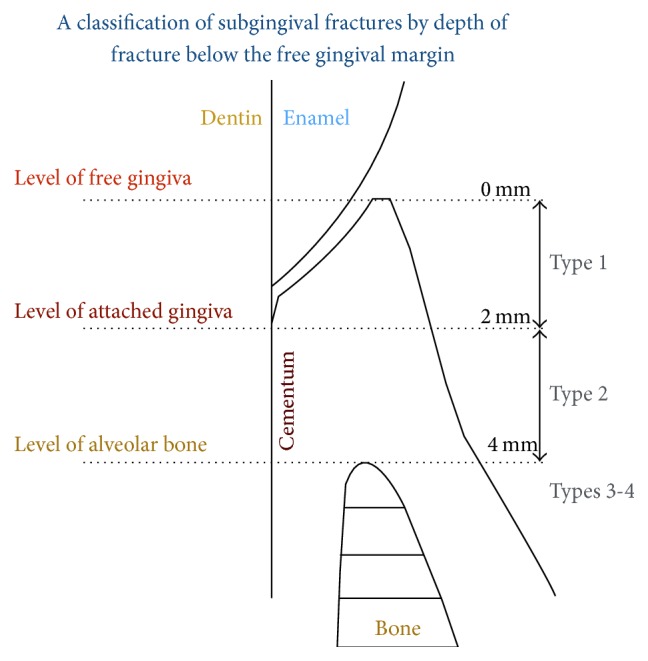


The position and the circumferential extent of the fracture are of considerable importance in treatment planning. However, the severity of the fracture in a subgingival direction is probably the most important factor influencing the treatment plan. With this respect, teeth with subgingival fractures may be classified into four categories [16] (Figure 1).

## 4. Diagnosis of Fractures

**Figure 2:**
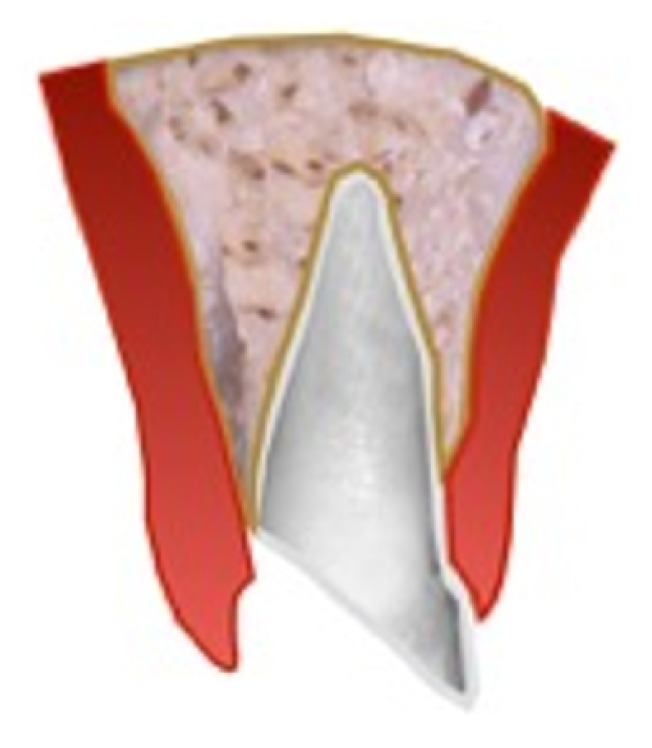


**Figure 3:**
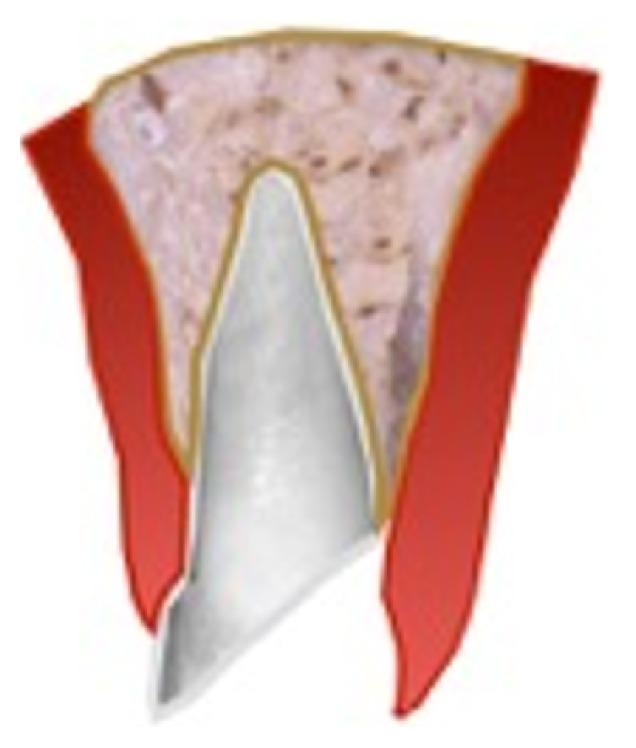


The first step in the process of determining if the teeth should be saved or extracted is to locate the most apical extent of the fracture. Traumatically fractured maxillary anterior teeth generally have an oblique fracture angle with the most apical portion located on the palatal or on the labial depending on the impact direction. For a palatal fracture the cause is usually the external direct force resulting from the impact on the maxillary central, whereas for a labial fracture the cause is the lower incisor impacting on the palatal of the upper incisor [17]. Often the extent and acuteness of the fracture angle may be challenging to identify where it ends radiographically. Thus, it is vital to locate the extent of the fracture clinically (Figures 2 and 3). Though it has been shown in the literature that one of the most important factors in the predictable restoration of endodontically treated teeth is having an adequate ferrule [18, 19]. The following are also significant factors which determine the treatment option and the prognosis of the fractured tooth: patient's age, dental eruption and stage of the root formation, location of the line fracture: palatal or labial, location of the line fracture in relation to the biological width, alveolar bone fracture, pulp exposition or proximity to the pulp, periodontal involvement, soft-tissue injuries, presence/absence of fractured tooth fragment, amount of remaining tooth structure, secondary traumatic injuries, occlusion, and aesthetics [3, 5].

## 5. Treatment of Fractures 

Treatments of fractures are often interdisciplinary [3]. In fact, CRF always involve periodontal tissue, external tooth structures (enamel and dentine), and sometimes pulpal tissues. Therefore, treatment alternatives can be divided into six different approaches:

1. Endotreatment + fragment reattachment
2. Endotreatment + periodontal surgery + fragment reattachment
3. Endotreatment + orthodontic extrusion + restoration of the teeth
4. Fragment removal and direct or indirect restoration
5. Endotreatment + post and core + periodontal surgery + prosthetic restoration
6. Extraction + implant

## 6. Case Reports

### 6.1. Case 1

**Figure 4:**
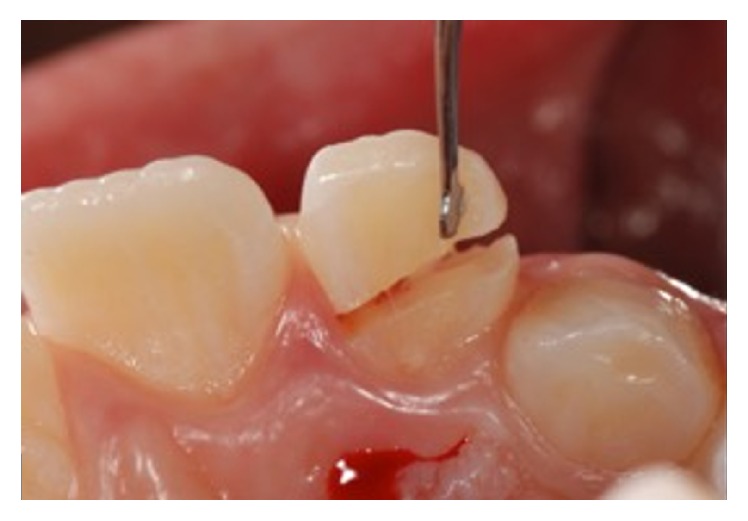


**Figure 5:**
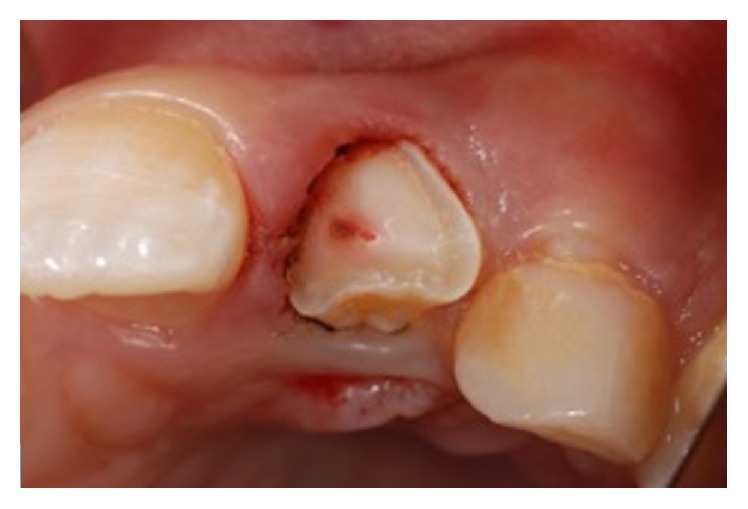


**Figure 6:**
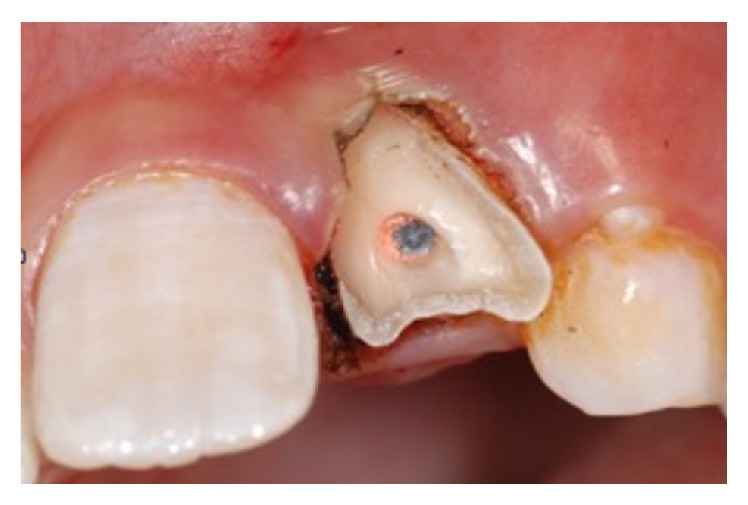


**Figure 7:**
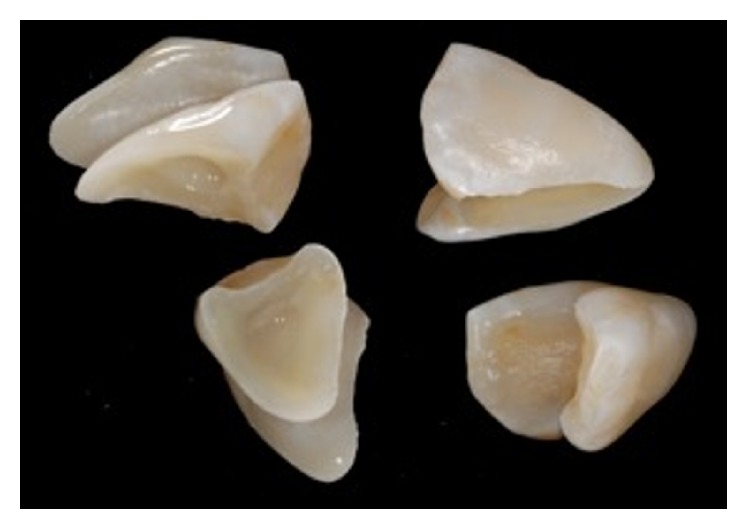


**Figure 8:**
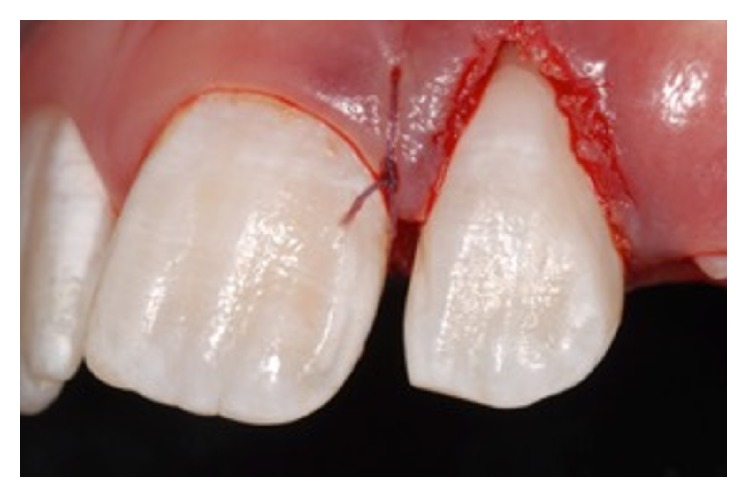


**Figure 9:**
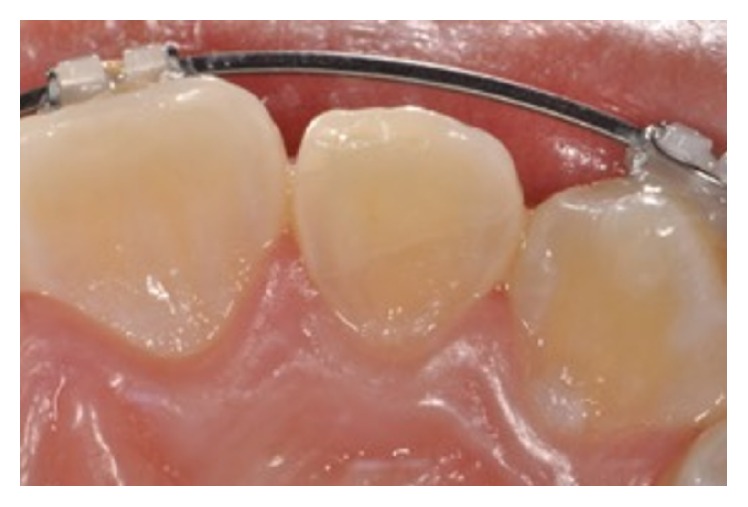


**Figure 10:**
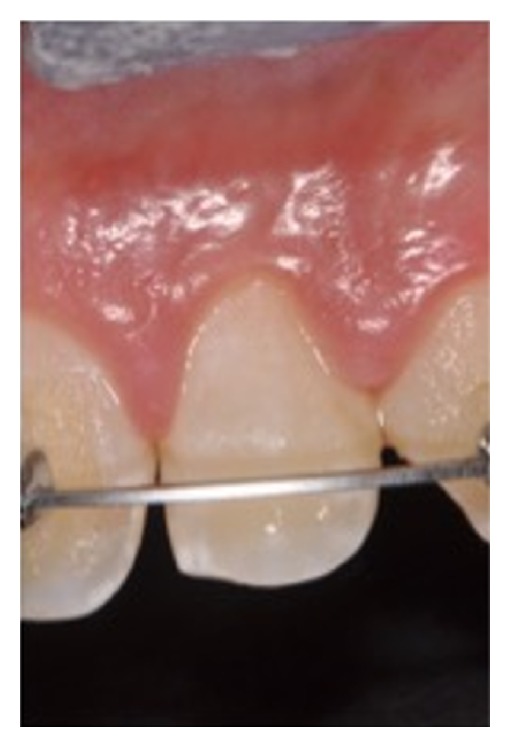


**Figure 11:**
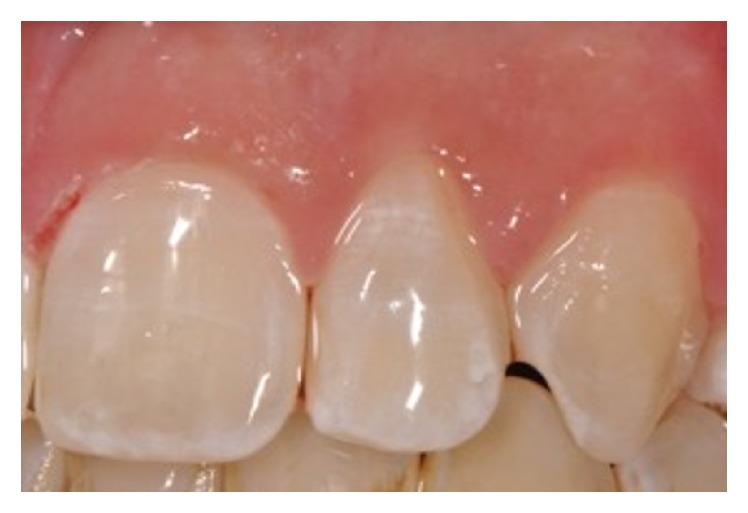


An 11-year-old patient presented following a complicated CRF trauma to tooth 2.2 (Figure 4). The treatment involved root canal treatment since there was pulp exposure (Figure 5). Due to the subgingival extent of the labial fracture line a flap was raised to expose the fracture margin (Figure 6). Following the root canal therapy and correct isolation, the fractured fragment (Figure 7) was cleaned, etched, and adhesively recemented using a heated composite, and then the flap was sutured (Figure 8). A few years later orthodontic therapy was carried out to resolve orthodontic problems, and the reattached fragment was still in place, showing normal periodontal maturation of the tissues and aesthetic and restorative integration (Figures 9 and 10). Five years since the initial treatment the restoration continues to maintain successful functional, aesthetic, and biologic parameters (Figure 11).

### 6.2. Case 2

**Figure 12:**
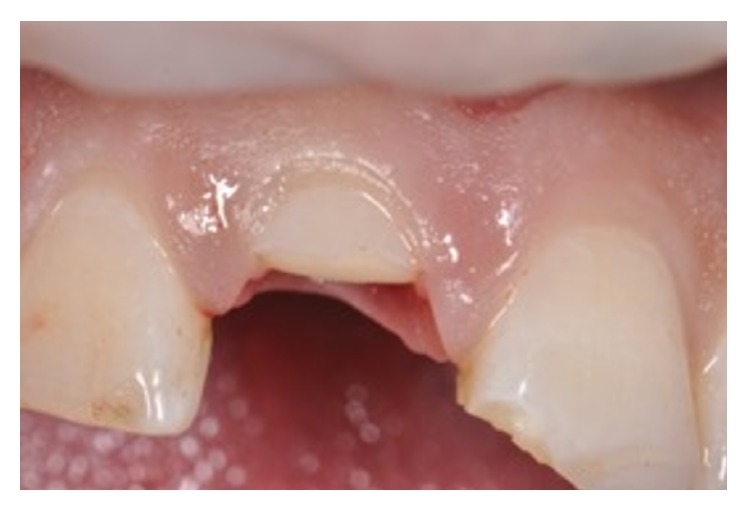


**Figure 13:**
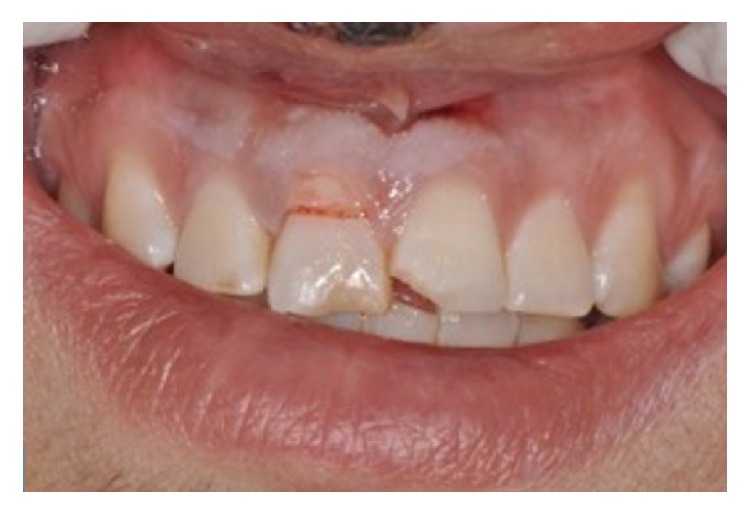


**Figure 14:**
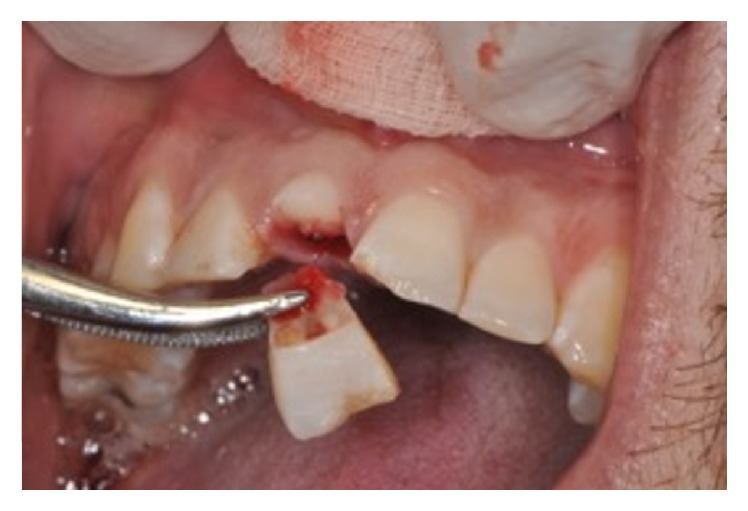


**Figure 15:**
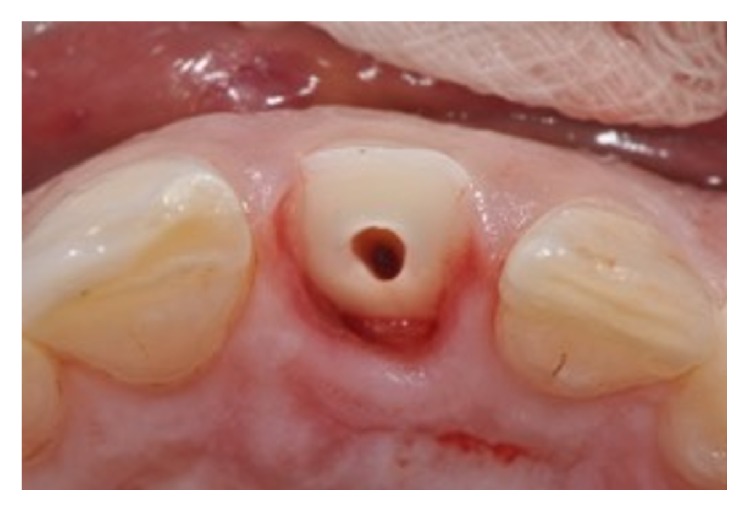


**Figure 16:**
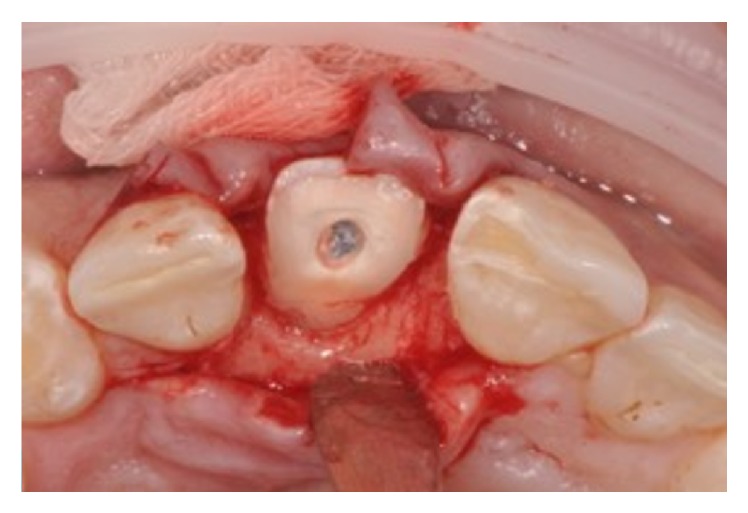


**Figure 17:**
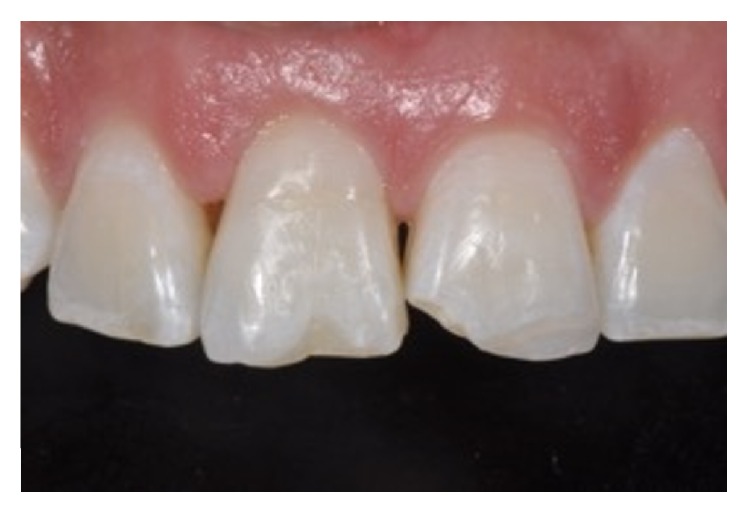


**Figure 18:**
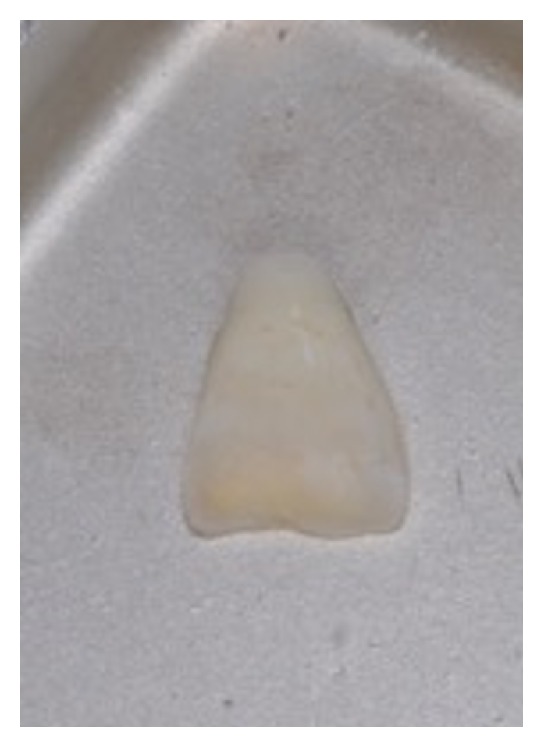


**Figure 19:**
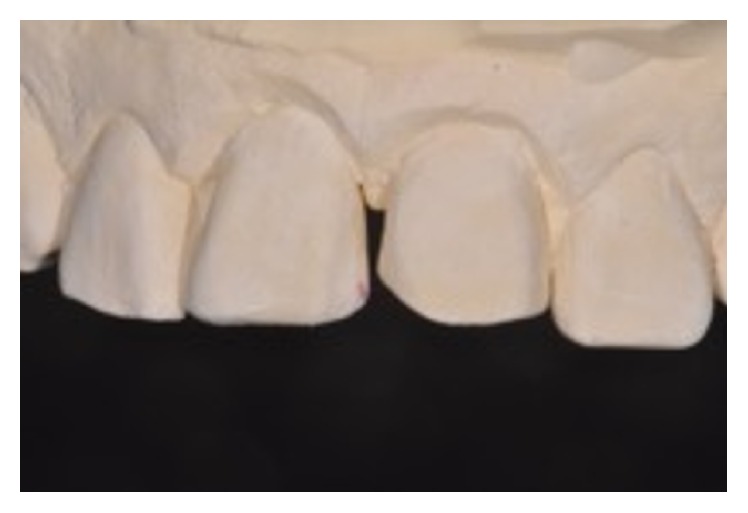


**Figure 20:**
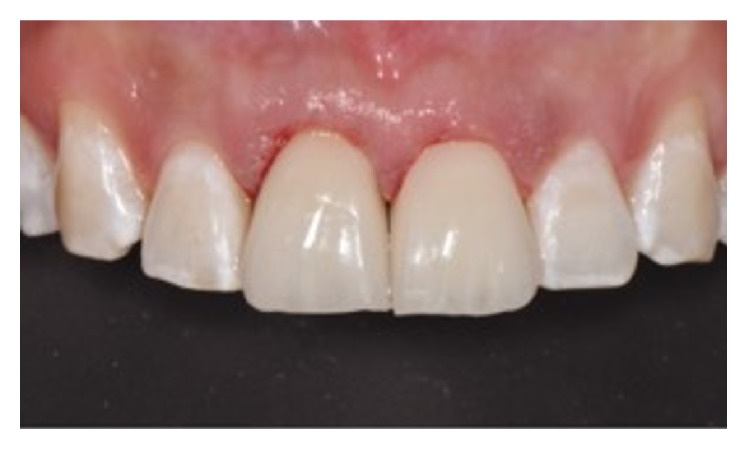


**Figure 21:**
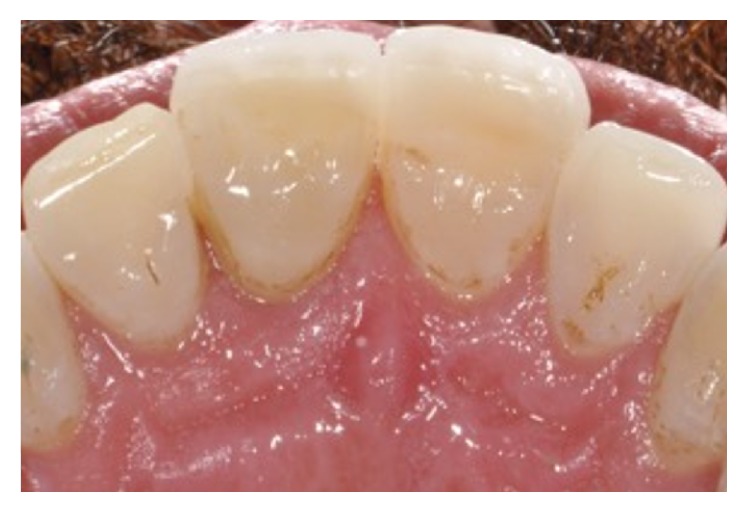


A 28-year-old man presented following a complicated CRF trauma of the 11 and less severe dentin-enamel fracture of the 21 (Figures 12 and 13). Removal of the fragment of the 11 revealed an almost complete detachment of the clinical crown with a deep palatal fracture line (Figures 14 and 15). Root canal therapy was required (Figure 16) and a flap was raised to surgically expose the palatal fracture margin through osteotomy (Figure 17). Following root canal therapy the tooth fragment was prepared with mechanical retention, cleaned, etched (Figure 18), and adhesively recemented using a heated composite. Once gingival healing was obtained, three months after trauma, it was decided to restore the two teeth with minimal preparation porcelain veneers to strengthen the labial surfaces (Figure 19), as well as to improve aesthetics in masking the labial fracture of the two teeth (Figure 20) which were placed (Figure 21), and after 18 months the papilla had reached full maturation and optimum aesthetic result (Figure 22).

## 7. Discussion

Conventional approaches to rehabilitating fractured anterior teeth include composite restorations and post-core-supported prosthetic restorations when the tooth has had pulpal exposure and extensive fracture of the crown [1, 3, 4]. The fractured segment is usually removed and post-core and crown restoration is done after root canal therapy. However, disadvantages of these two alternatives may be the reduced aesthetic results (both immediately and in the long term) due to discoloration of composite resin restorations and aggressiveness of post-core full crowns. The use of tooth fragment reattachment technique to preserve the fractured segment of a tooth has been in the literature for decades [11, 20] and offers better short- [21, 22] and medium-term [23] results compared to resin composite restorations. This technique is more so encouraged nowadays due to the improvement of newer adhesives and especially in the case of younger patients. It is an optimal approach for restoring fractured anterior teeth, when the fragment is available [7, 10, 23]. The fractured fragment has been proposed as a favorable crown repair material due to its superior morphology, conservation of structure, and patient acceptance [10]. It requires minimal preparation of the tooth, is more esthetic and faster to reattach than a composite resin restoration, and has a psychological benefit to the patient that his own tooth has been retained. This is regardless as to whether root canal treatment is required or not. Loss of vitality followed by proper endodontic therapy proved to affect tooth biomechanical behavior only to a limited extent. Whether it is because of caries or restorative procedures, tooth strength is reduced in proportion to coronal tissue loss. Therefore, the key strategies to restore endodontically treated teeth are to minimize the removal of tooth structure, especially in the cervical region to maximize the ferrule effect, to use adhesive procedures at both radicular and coronal levels to strengthen remaining tooth structure, and to optimize restoration stability and retention and use post and core materials with physical properties similar to those of natural dentin, because of the limitations of current adhesive procedures [24]. The concepts that support this therapeutic option are similar to that of endocrowns [25, 26] but with the original tooth fragment as the ideal material, avoiding the use of artificial materials which require further tooth demolition and preparation to obtain mechanical retention, deep posts, and ferrule for conventional restorations. The use of cast metal cores was associated with wedge effect which may lead to tooth fracture [19], whereas adhesion of prefabricated posts has limited long-term stability [27, 28]. Alternatively, maintaining as much enamel as possible is an advantage when using endocrowns, porcelain veneers, or tooth fragments due to increased bond strength of adhesives on enamel [8, 9]. In both case reports showed in this manuscript, conventional treatment may have led to post-core crowns or even extraction and implant placement. But in making the more conservative choice of treatment, the authors have taken into consideration other important factors like the patient's age, the irretrievability of the restoration in case of failure, and the possibility of postponing more aggressive treatment without any negative implications in the meantime. The key to this type of treatment was to immediately expose the fracture margin to allow an ideal isolation with rubber dam placement. In the first case the depth of the labial fracture margin presented an esthetic challenge; however given the young age of the patient and the incomplete eruptive phase of the tooth and gingiva, following a minimal gingivectomy and osteotomy, the cementation of the fragment and resulting aesthetics were obtained satisfactorily, whereas, in the second case, the palatal location of the deep fracture margin permitted the exposure with a palatal flap elevation and osteotomy and minimal labial involvement of the gingival tissues and papillae. Subsequently, given the patients age, it was decided to further increase the esthetic final result and the ferrule effect and thus the durability of the treatment with porcelain veneers [29, 30].

## 8. Conclusion

The authors consider the immediate reattachment of the tooth fragment not a temporary or transitory alternative but a reliable and long-term treatment alternative, considering the efficacy of current adhesive systems. This type of treatment is immediate, uses the ideal restorative material, and eliminates the need for aggressive and complex restorations [30, 31].

## Figures and Tables

**Figure 22:**